# Electrospun Nanofibers as Carriers of Microorganisms, Stem Cells, Proteins, and Nucleic Acids in Therapeutic and Other Applications

**DOI:** 10.3389/fbioe.2020.00130

**Published:** 2020-02-25

**Authors:** Spase Stojanov, Aleš Berlec

**Affiliations:** ^1^Department of Biotechnology, Jožef Stefan Institute, Ljubljana, Slovenia; ^2^Faculty of Pharmacy, University of Ljubljana, Ljubljana, Slovenia

**Keywords:** nanofibers, electrospinning, bacteria, fungi, viruses, stem cells, proteins, DNA

## Abstract

Electrospinning is a technique that uses polymer solutions and strong electric fields to produce nano-sized fibers that have wide-ranging applications. We present here an overview of the use of electrospinning to incorporate biological products into nanofibers, including microorganisms, cells, proteins, and nucleic acids. Although the conditions used during electrospinning limit the already problematic viability/stability of such biological products, their effective incorporation into nanofibers has been shown to be feasible. Synthetic polymers have been more frequently applied to make nanofibers than natural polymers. Polymer blends are commonly used to achieve favorable physical properties of nanofibers. The majority of nanofibers that contain biological product have been designed for therapeutic applications. The incorporation of these biological products into nanofibers can promote their stability or viability, and also allow their delivery to a desired tissue or organ. Other applications include plant protection in agriculture, fermentation in the food industry, biocatalytic environmental remediation, and biosensing. Live cells that have been incorporated into nanofibers include bacteria and fungi. Nanofibers have served as scaffolds for stem cells seeded on a surface, to enable their delivery and application in tissue regeneration and wound healing. Viruses incorporated into nanofibers have been used in gene delivery, as well as in therapies against bacterial infections and cancers. Proteins (hormones, growth factors, and enzymes) and nucleic acids (DNA and RNA) have been incorporated into nanofibers, mainly to treat diseases and enhance their stability. To summarize, incorporation of biological products into nanofibers has numerous advantages, such as providing protection and facilitating controlled delivery from a solid form with a large surface area. Future studies should address the challenge of transferring nanofibers with biological products into practical and industrial use.

## Introduction to Nanofibers

Nanotechnology includes the use of nanomaterials and represents one of the newest approaches in medicine, and also in science in general. Nanomaterials can have useful physicochemical properties, can be biocompatible and non-toxic, and can sense and respond to environmental stimuli (Shahriar et al., 2019). Nanofibers are an example of nanomaterials that are mostly produced by electrospinning (Agrahari et al., 2017). This is a method in which polymers in solution are formed into nano-sized continuous fibers by applying a strong electric field. The polymer solution is squeezed from a syringe into a drop. High electrostatic forces overcome the cohesive forces, which results in formation of the jet, its continuous elongation under a whipping motion, evaporation of the solvent, and formation of the nano-sized fibers (Teo and Ramakrishna, 2006; Bhattarai et al., 2018; Shahriar et al., 2019). Different compounds can be added to the polymer solution, and thus become incorporated into the nanofibers during this process. Nanofibers have applications in several different areas, such as health care (e.g. tissue engineering, regenerative medicine, wound dressing, drug delivery), energy storage, and environmental remediation (Kenry and Lim, 2017).

Nanofibers can be produced from various polymeric materials, which can be natural, synthetic, or a combination of both. Natural polymers have better biocompatibility and lower immunogenicity. On the other hand, synthetic polymers offer greater flexibility in their synthesis and modification (Hu et al., 2014).

Electrospinning techniques can be divided into six categories: basic electrospinning; blend electrospinning; coaxial electrospinning; emulsion electrospinning; melt electrospinning; and gas jet electrospinning (Shahriar et al., 2019). Basic electrospinning is performed with a single polymer, while blend electrospinning involves mixing polymers and altering their ratios in the blend. For incorporation of drugs for delivery, blend electrospinning can improve the tunability of the drug-loaded fibers, and allow for controlled drug release (Tipduangta et al., 2016). The release of the drug from coaxially electrospun nanofibers begins with an initial burst release from the sheath that is followed by a long period of sustained release from the core (Lu et al., 2016). In emulsion electrospinning, the drugs and surfactants form either water-in-oil or oil-in-water emulsions. This type of electrospinning uses a single nozzle, and allows direct formation of core–shell structured nanofibers that can better protect the encapsulated materials from environmental conditions (Zhang et al., 2018). In melt electrospinning, a polymer melt is used instead of a polymer solution. Nanofibers from polymer melts have demonstrated slow drug release without a burst phase, while high drug release with a burst phase is seen for nanofibers from polymer solutions (Lian and Meng, 2017). Gas jet electrospinning combines electrospinning with a gas jet device, in which the capillary of the spinning fluid is surrounded by a tube with a gas jet, which promotes the formation of finer and more uniform nanofibers (Lin et al., 2008).

**FIGURE 1:**
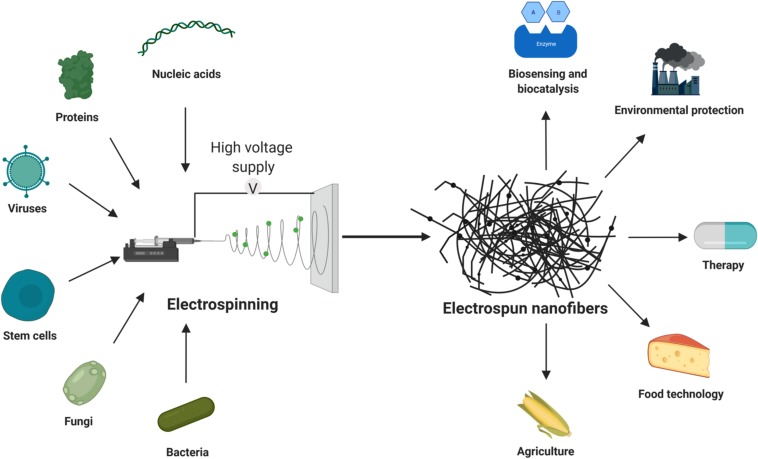
Incorporation of living cells, proteins and nucleic acids into electrospun nanofibers, and their applications.

This review will provide a brief overview of nanofibers with incorporated live cells, viruses, proteins and nucleic acids. Polymers that are used to make these nanofibers will be reviewed, as well as their applications (Figure 1). For other types and applications of nanofibers, the reader is referred elsewhere (Kenry and Lim, 2017; Min et al., 2018).

## Biological Products in Nanofibers

The biological products referred to here include live cells and viruses, as well as their components, such as proteins and nucleic acids. Biological products are mainly intended for use in medicine (e.g. as biopharmaceuticals), but can also be used in agriculture and the food industry, and for environmental protection, biosensing, and biocatalysis. They have low stability and degrade rapidly when administered to the human host. Their low viability or stability can be overcome by the use of electrospun nanofibers. Incorporation of biological products into nanofibers can protect them from environmental conditions, and allow their controlled release (Sebe et al., 2015).

Biopharmaceuticals are highly effective drugs and have great therapeutic potential against various diseases, such as cancers, diabetes, Crohn’s disease, rheumatoid arthritis, and many others. Among the 15 best-selling drugs in 2018, 11 were biopharmaceuticals.^1^ However, therapies with biopharmaceuticals also have their drawbacks, such as limited cell viability, short half-life after administration (Laflamme et al., 2007), immunogenicity, low stability, and degradation (Akash et al., 2015). One of the ways to overcome these issues is to incorporate the biopharmaceuticals into nanofibers. This can allow them to remain stable or viable (Xavier et al., 2016), improve their pharmacokinetic properties, and reduce their immunogenicity (Walsh et al., 2003). Nanofibers therefore represent a safe and effective alternative approach for delivery of biologicals.

To date, several types of biological products have been included in nanofibers to improve their delivery in medical applications (Figure 1). Among the cells used, probiotic bacteria have been incorporated most often (Heunis et al., 2010; Akbar et al., 2018; Skrlec et al., 2019; Zupancic et al., 2019). Cyanobacteria and fungus are also incorporated and they have therapeutic, agricultural and environmental properties. Nanofibers have been used as scaffolds for stem-cell delivery, mainly for tissue engineering and wound healing (Shabani et al., 2009; Gizaw et al., 2019). Nanofibers with viruses have mainly been used for gene delivery, while bacteriophages and oncolytic viruses have been applied against some pathogenic bacteria and cancers respectively (Lee et al., 2011; Nogueira et al., 2017). Nucleic acids have been incorporated into nanofibers for gene delivery, usually in the form of plasmid DNA (Li et al., 2013). RNA has also been incorporated into nanofibers for gene delivery, although the main role here has been gene silencing (Diao et al., 2015). Proteins that have been incorporated in nanofibers have mainly been hormones, growth factors, and enzymes.

## Properties and Polymer Composition of Nanofibers Containing Biological Products

Nanofibers properties differ in their surface-to-volume ratio, porosity, morphology and shape. These properties can be modified and changed to adjust the suitability of the polymer for its intended application. The properties of nanofibers are influenced by electrospinning parameters (i.e. electric field applied, distance between needle and collector, flow rate, needle diameter), solution parameters (i.e. solvent, polymer concentration, viscosity, conductivity), and environmental parameters (i.e. humidity, temperature).

Increasing the applied voltage during the formation of nanofibers increases their diameter (Haider et al., 2018). A higher flow rate of the polymer through the syringe can result in the formation of beads and an increased pore size. Increased distance between the metallic needle tip and the collector thins the jet and reduces the average fiber diameter (Robb and Lennox, 2011). Low polymer concentrations can cause the polymer chain to break before reaching the collector, while higher concentrations of the polymer can result in drying of the solution at the tip of the needle (Haider et al., 2018). The conductivity of the solution can impact upon the jet formation and the diameter of the nanofibers, and solutions with very high or very low conductivity cannot be electrospun (Angammana and Jayaram, 2011). The shape and morphology of the fibers depend on the glass transition temperature, solubility, and composition of the substrates used, and on the molecular weight of the polymer. Electrospinning is also affected by the viscoelasticity, concentration, surface tension, and electrical conductivity of the polymer solution, and also by the humidity, temperature, and vapor pressure of the solvent (Bhattarai et al., 2018). In addition, biological products can affect the properties of the nanofibers. For example, adding *Lactobacillus plantarum* into polyethylene oxide (PEO) solutions increases the conductivity due to the extracellular proteins and ions introduced with the probiotics (Skrlec et al., 2019). Furthermore, different concentrations of bacteria can increase the viscosity of the polymer solution (Zupancic et al., 2019).

The delivery rate of the biological products depends on their distribution within the nanofibers and on the nature of the polymer. The hydrophilic/hydrophobic properties of polymers can affect the release profile. In emulsion electrospinning, co-polymerization of hydrophobic polymers with hydrophilic polymers affects the drug distribution (Vlachou et al., 2019). Core–shell electrospinning can also slow down the release rate, due to the presence of the shell, which can also prevent the burst release of drugs or cells. The thicker the core, the more time it will take for the drug/cells to be released from the electrospun nanofibers (Yang et al., 2019).

Polymers for the formation of nanofibers can be classified on the basis of their origin, as natural or synthetic. For the delivery of biological products, synthetic polymers have been used more often. Also, several natural polymers that have been used for nanofiber production have low mechanical strength and stability. This is why it is often necessary to incorporate additional synthetic or natural polymers into nanofibers, to obtain the required properties (DeFrates et al., 2018).

## Polymers

### Natural Polymers

Polysaccharides and proteins are the most common natural polymers that are used in electrospinning and production of nanofibers for the delivery of biological products. Among the polysaccharides, chitosan, cellulose, and alginate derivatives have the potential to be electrospun into nanofibers and to serve as a delivery vehicle.

Chitosan is a linear co-polymer of *N*-acetyl-D-glucose amine and D-glucose amine. Due to its polycationic nature, chitosan can interact with negatively charged molecules, such as proteins, anionic polysaccharides, fatty acids, bile acids, and phospholipids (Ahmed and Ikram, 2016). The free amino groups of chitosan can neutralize gastric acid and form a protective barrier in the stomach; this suggests that chitosan is an ideal material for incorporation of live organisms and molecules that are sensitive to acidic environments. Nevertheless, the polycationic nature and rigid chemical structure of chitosan limits its electrospinnability. The formation of nanofibers is facilitated by blending chitosan with another polymer, thereby allowing the formation of hydrogen bonds (Cázarez et al., 2018). Chitosan has been combined with PEO, poly (lactic-co-glycolic acid) (PLGA), and polyacrylamide to form nanofibers, which have served as delivery systems, respectively, for *Bacillus* sp. 25.2.M (Zupancic et al., 2018), *Trichoderma viride* (Spasova et al., 2011), and mesenchymal stem cells (MSCs) (Kim Y. C. et al., 2016).

Cellulose is a polysaccharide of the plant cell wall. Cellulose nanofibers have great mechanical stiffness and strength; however, their structure is porous, and without the protection of an outer shell, the incorporated cells or molecules are rapidly released into the environment. This has been prevented by oxidation of the hydroxy groups into carboxylic groups using 2,2,6,6-tetramethylpiperidine-1-oxyl-radical–mediated oxidation, and the cellulose nanofibers thus produced allowed sustained release of *Lb. plantarum* cells into the desired region of the intestinal tract (Luan et al., 2018). Cellulose also has limited melting and solubility in organic solvents, which was overcome by using cellulose acetate (Khoshnevisan et al., 2018). Cellulose acetate nanofibers were used as the scaffold material for formation of *Lb. plantarum* biofilms, and showed high stability and good gastrointestinal resistance (Hu et al., 2019). The combination of cellulose acetate with the synthetic polymer polyvinyl alcohol (PVA) was used for the production of hybrid electrospun nanofibers for the encapsulation of fungus that provided aflatoxin B2 removal from contaminated water (Moustafa et al., 2017). Cellulose ethers, such as carboxymethyl cellulose and methylcellulose, have also been used in combination with synthetic polymers, for encapsulation of *Staphylococcus epidermidis* (Kurecic et al., 2018) and lysozyme (Yang et al., 2008).

Alginate is an anionic polysaccharide that is widely distributed in the cell walls of brown algae. It is a linear unbranched polysaccharide that contains different amounts of (1→4′)-linked β-D-mannuronic acid and α-L-guluronic acid residues. Alginate and its derivatives are biodegradable and have controllable porosity; however, electrospinning of pure sodium alginate is difficult. The addition of another polymer has therefore been necessary to control the viscosity and spinnability of alginate. Together with PVA, sodium alginate has been electrospun into nanofibers that have served as delivery systems for insulin (Sharma et al., 2013), and for *Lactobacillus rhamnosus* GG in food technology (Ceylan et al., 2018).

Fructo-oligosaccharides occur naturally in plants and are composed of linear or branched chains of 2–60 fructose units. They are advantageous because they are non-sweet, calorie free, and non-cariogenic, and they are considered as soluble dietary fiber (Sabater-Molina et al., 2009). Fructo-oligosaccharides (2.5%; w/w) have been combined with PVA to encapsulate *Lb. plantarum* (Feng et al., 2018).

Proteins are the second most abundant natural polymers used to make nanofibers loaded with biological products. Proteins can attach to live cells, due to their native cell attachment motifs (DeFrates et al., 2018). The most common proteins that are electrospun for drug delivery applications are fish sarcoplasmic proteins and the collagen, elastin, and silk proteins.

Sarcoplasmic proteins are found in the sarcoplasm or fluid surrounding the myofibrils. Fish sarcoplasmic proteins from the Atlantic cod (*Gadus morhua*) are elongated polymers that have fiber diameters from hundreds of nanometers to a few micrometers. Together with the potential health benefits of consuming fish, these characteristics make fish sarcoplasmic proteins an attractive material for electrospinning of nanofibers. Additionally, they have been shown to inhibit the enzyme dipeptidyl peptidase-4, which is responsible for glucose metabolism and is linked to type 2 diabetes (Li-Chan et al., 2012). Thus these nanofibers can be used for the delivery of antidiabetic drugs, such as insulin (Stephansen et al., 2015a).

Collagen is the main structural protein of the extracellular matrix, as it provides structural support for the cells and biological and mechanical stability to the tissue. Poly (ε-caprolactone-co-ethyl ethylene phosphate) (PCLEEP) and collagen hybrid nanofibers have been used for effective sustained delivery of neurotrophin-3 and micro-RNA (miRNA)-222 (Nguyen et al., 2017).

Elastin is also present in the extracellular matrix, and maintains the elasticity of the connective tissue in the human body. Like collagen, it is of interest in medicinal applications, such as drug delivery and tissue engineering. Tropoelastin is a soluble precursor of elastin, and it is composed of hydrophobic and hydrophilic crosslinking domains. Elastin−like polypeptides are biopolymers that are composed of amino-acid sequences found in the hydrophobic domain of tropoelastin (MacEwan and Chilkoti, 2010). Their temperature phase transition properties allow controlled drug delivery; however, pure elastin−like polypeptides are not suitable for electrospinning of nanofibers due to their low mechanical strength. To improve their mechanical properties and allow electrospinning, elastin−like polypeptides can be blended with poly (ε-caprolactone) (PCL). Such hybrid nanofibers have served as carriers for adeno-associated virus for gene delivery (Lee et al., 2011).

Silk proteins represent a unique family of natural fibrous biopolymers that are produced by some insects, spiders, and worms. They are promising materials for drug delivery and tissue engineering due to their biocompatibility, slow biodegradability, self-assembly, and controllable structure and morphology (Elzoghby et al., 2015). There are two main silk proteins from the silkworm (*Bombyx mori*): sericin (sticky protein, on the outside of silk strands), and fibroin (silk strand core protein; used in electrospinning). The combination of silk fibroin and PEO electrospun nanofibers has served as a delivery system for epidermal growth factor (EGF). The ultra-fine porous structure of the nanofibers allowed oxygen permeation, promoted drainage of fluids, and inhibited exogenous microorganism invasion (Schneider et al., 2009).

As well as proteins and carbohydrate polymers, other types of natural polymers have been used as materials for electrospinning of nanofibers. Poly (hydroxybutyrate) (PHB) is natural polyester that belongs to the class of polyhydroxyalkanoates and is produced by a wide range of microorganisms (Kuntzler et al., 2018). PHB is a fully biodegradable and biocompatible polymer that can be blended with other synthetic or natural polymers to improve its properties (Bonartsev et al., 2007). PHB and PCL solutions have been combined to make electrospun nanofibers for incorporation of *Spirulina* sp. LEB 18 (Schmatz et al., 2016).

### Synthetic Polymers

Synthetic polymers predominate in the production of nanofibers that contain biological products. The most common synthetic polymers for the production of nanofibers are: PEO, PVA, PCL and its co-polymers, polyvinylpyrrolidone (PVP), and poly lactic acid (PLA) (including its enantiomeric forms, poly L-lactic acid and poly D-lactic acid). All of these have been approved by the US Food and Drug Administration as drug delivery systems or as scaffolds for tissue engineering (Mansour et al., 2010). These polymers can be used alone or in combination with other synthetic and/or natural polymers.

Polyethylene oxide is a neutral, non-toxic, biocompatible and hydrophilic polymer that is widely used in drug delivery and tissue engineering. It is not absorbed in the gastrointestinal tract, shows weak immunogenicity, and good compatibility with live cells. Poly (ethylene glycol) (PEG) is also a polymer of ethylene oxide, and differs from PEO only in its molecular weight. The combination of these polymers with other synthetic and natural polymers can enhance their mechanical and physical properties (Bhattarai et al., 2018).

Polyvinyl alcohol is another water-soluble synthetic polymer. Due to its high hydrophilicity and good solubility in water, PVA has limited applicability for electrospinning of nanofibers. To improve its mechanical properties and water resistance, PVA has to be modified by either chemical or physical crosslinking (Park et al., 2010).

Polyvinylpyrrolidone is a hydrophilic polymer made from *N*-vinylpyrrolidone monomers by free-radical polymerization. PVP is very hygroscopic and can absorb up to 40% water by weight (Kariduraganavar et al., 2014), which causes problems for nanofiber production. Coaxial electrospinning can be used to improve the moisture resistance of the nanofibers and to make them more durable and flexible (Letnik et al., 2015).

Polycaprolactone is another biocompatible and biodegradable polymer that has a low melting point and glass transition temperature. PCL is highly soluble in organic solvents, such as hexafluoroisopropanol, dichloromethane, chloroform, methanol, tetrahydrofuran, and others (Rameshbabu et al., 2018); it is therefore more suitable when longer degradation times are required. PCL variants with more hydrophilic monomers promote more rapid water diffusion, thus increasing the degradation rate (Guarino et al., 2017). To make nanofibers, co-polymers of PCL have mostly been used. Co-polymerization of PCL with poly (ethyl ethylene phosphate) forms PCLEEP, which is an amphiphilic co-polymer with good potential for drug delivery (Chew et al., 2005, 2007; Zhang et al., 2010; Nguyen et al., 2017). Nanofibers from co-polymers of PCL with polyethylenimine (PEI) have been used for immobilization of *Gluconobacter oxydans* (Gordegir et al., 2019). Nanofibers made of poly (ethylene carbonate) and PCL blends have been used to incorporate and deliver vascular endothelial growth factor (Zhang et al., 2012).

Poly lactic acid is biodegradable and biocompatible aliphatic polyester. It is considered bio-based polymer because of the natural origin of its monomer, lactic acid. However, PLA is synthesized by direct polymerization of lactic acid monomers or by ring-opening polymerization of cyclic lactide dimers, and is therefore classified as synthetic polymer. Its properties, such as crystallinity and thermal stability, depend on the D-/L- composition of lactic acid. In general, PLA has good mechanical and physical properties, such as high modulus, high strength, good clarity, and good barrier properties, which make it an appropriate candidate for nanofiber production, drug delivery, and tissue engineering (Nofar et al., 2019). Some limitations of PLA, such as low melting point and slow crystallization rate, can be overcome by blending PLA with another polymer. PLGA represents a physically strong and highly biocompatible co-polymer of PLA and poly glycolic acid. It is biodegradable, and has been extensively studied as a delivery vehicle for biologics. PLGA is more hydrophobic than PLA, which results in slower degradation (Makadia and Siegel, 2011). As a delivery vehicle for biological products, PLGA electrospun nanofibers are most commonly used for the delivery of stem cells.

Polymers of methacrylic acid and methylmethacrylate (commercially known as Eudragit L100) have also been used for electrospinning of nanofibers. The water solubility of these polymers is pH dependent, which allows pH-dependent drug release (da Silva et al., 2017). The biggest advantage of Eudragit L100 is that it protects the drug from the acidic environment of the stomach, and allows controlled release in the intestine and colon (Kaur et al., 2018).

## Bacteria, Fungi, Stem Cells, and Viruses in Nanofibers

### Bacteria

Bacteria that have been incorporated into electrospun nanofibers usually belong to the group of probiotics. Probiotics are live microorganisms that can confer health benefits on the host when administered in adequate amounts. Their low viability and high sensitivity to environmental factors represent a hurdle for their wider application. For this reason, there is a need for new probiotic delivery systems (Kim J. et al., 2016). Different electrospun nanofibers have been used for incorporation of probiotics, have allowed their viability to be maintained, and have provided for their delivery. Non-probiotic bacteria have also been incorporated into nanofibers, and apart from being used for therapeutic purposes, they have also been applied as biosensors and biocatalysts, and for food and agricultural purposes. Table 1, shows the bacteria that have been incorporated into different nanofibers, along with their applications.

**TABLE 1:** Systems for incorporation of bacteria into electrospun nanofibers, and their designed uses.

**Bacterium**	**Source code/strain**	**Nanofiber polymer**	**Purpose**	**References**
*Lb. plantarum*	ATCC 8014	Polyethylene oxide	Delivery system for probiotics	Skrlec et al., 2019
*Lb. plantarum*	423	Polyethylene oxide	Delivery system for bacteriocins and probiotics	Heunis et al., 2010
*Lb. plantarum*		Cellulose	Delivery system for probiotics	Luan et al., 2018
*Lb. acidophilus*		Soluble dietary fiber, oil-palm trunk + oil-palm fronds, with polyvinyl alcohol	Encapsulation of probiotic bacteria	Fung et al., 2011
*Lb. acidophilus*		Polyvinyl alcohol + polyvinyl pyrrolidone	Delivery system for probiotics (bacterial vaginosis)	Nagy et al., 2014
*B. lactis*	Bb12	Polyvinyl alcohol	Encapsulation of probiotic bacteria	Akbar et al., 2018
*S. thermophilus*	TH-4			
*Lb. paracasei*	431			
*B. animalis*	Bb12	Polyvinyl alcohol	Encapsulation of probiotic bacteria	Lopez-Rubio et al., 2009
*Lb. plantarum*		Fructo-oligosaccharides + Polyvinyl alcohol	Encapsulation of probiotic bacteria	Feng et al., 2018
*Lb. acidophilus*		Chitosan	Encapsulation of probiotic bacteria	Ebrahimnejad et al., 2017
*Lb. acidophilus*	ATCC 4356	Polyethylene oxide	Encapsulation of probiotic bacteria	Zupancic et al., 2019
*Lb. delbrueckii* ssp. *bulgaricus*	ATCC 11842			
*Lb. casei*	ATCC 393			
*Lb. gasseri*	ATCC 33323			
*Lb. paracasei*	ATCC 25302			
*Lb. plantarum*	ATCC 8014			
*Lb. reuteri*	ATCC 55730			
*Lb. rhamnosus*	ATCC 53103			
*Lb. salivarius*	ATCC 11741			
*L. lactis* ssp. *cremoris*	MG1363			
*Lb. plantarum*	CICC 23941	Cellulose acetate	Food technology (starter culture)	Hu et al., 2019
*Lb. rhamnosus*	GG	Polyvinyl alcohol/sodium alginate	Food technology (food preservation)	Ceylan et al., 2018
*Lb. gasseri*		Polyvinyl alcohol	Food technology (food supplements)	Amna et al., 2013
*Bacillus* sp.	25.2.M	Polyethylene oxide/chitosan + polyethylene oxide	Delivery system for probiotics (periodontal disease)	Zupancic et al., 2018
*S. epidermidis*		Carboxymethyl cellulose/Polyethylene oxide	Delivery system for probiotics (diabetic foot)	Kurecic et al., 2018
*G. oxydans*	DSM2343	Polycaprolactone/polyethyleneimine	Biosensing (glucose)	Gordegir et al., 2019
*P. agglomerans*	ISIB55	Polyvinyl alcohol	Agriculture (seed coating)	De Gregorio et al., 2017
*B. caribensis*	ISIB40			
*Spirulina* sp.	LEB 18	Poly hydroxybutyrate–hydroxyvalerate/polycaprolactone	Tissue engineering scaffold	Schmatz et al., 2016
*Spirulina* sp.		Polyethylene oxide	Tissue engineering scaffold	de Morais et al., 2010
*Spirulina* sp.	LEB 18	Poly hydroxybutyrate	Food packaging	Kuntzler et al., 2018

*Lactobacillus* is the most common genus of probiotic bacteria that has been incorporated into nanofibers. *Lactobacillus acidophilus* is present in the normal vaginal flora, and it can prevent colonization and proliferation of pathogenic microorganisms. Successful incorporation of *Lb. acidophilus* in PVA and PVP nanofibers can enhance the viability of this bacterium for up to 90 days, and has been suggested as an easy-to-use dosage form against bacterial vaginosis (Nagy et al., 2014). The viability of *Lb. acidophilus* has been increased for 78.6 – 90% when incorporated into agrowaste-based nanofibers combined with PVA; i.e. soluble dietary fiber from okara (soybean solid waste), oil-palm trunks, and oil-palm fronds (Fung et al., 2011). Several probiotic bacteria can produce antibacterial substances that are active against pathogenic bacteria. Plantaricin 423 is a bacteriocin that is produced by *Lb. plantarum* 423. Incorporation of bacteriocin-producing cells into electrospun PEO nanofibers represents a drug-delivery system for bacteriocins and probiotic lactic acid bacteria (Heunis et al., 2010). *Lb. plantarum* ATCC 8014 was successfully incorporated into PEO nanofibers in combination with the lyoprotectants sucrose and trehalose, and its long term viability was demonstrated (i.e. up to 6 months) (Skrlec et al., 2019). Like *Lb. acidophilus*, *Lb. plantarum* has also been incorporated into natural polymers. Cellulose nanofibers prepared with the 2,2,6,6-tetramethylpiperidine-1-oxyl radical–mediated oxidation system showed sustainable release of viable *Lb. plantarum* cells, and were suggested as a system for probiotic delivery into the intestinal tract (Luan et al., 2018). As well as viability, incorporation into nanofibers can enhance the thermal stability of bacteria. This has been investigated through the incorporation of *Lb. plantarum* into nanofibers made from fructo-oligosaccharides and PVA, which resulted in significantly enhanced *Lb. plantarum* survival under moist heat treatment (Feng et al., 2018). The damaging effects of the gastrointestinal environment on orally administered probiotics have been mitigated by their incorporation into chitosan nanofibers that has provided increased viability and stability of *Lb. acidophilus* in simulated gastric and intestinal environments (Ebrahimnejad et al., 2017). Recently, nine species of lactobacilli (i.e. *Lb. acidophilus*, *Lb. delbrueckii* ssp. *bulgaricus*, *Lb. casei*, *Lb. gasseri*, *Lb. paracasei*, *Lb. plantarum*, *Lb. reuteri*, *Lb. rhamnosus*, *Lb. salivarius*), together with *Lactococcus lactis* were compared for their incorporation into PEO nanofibers. All of these species remained viable after their incorporation, and their viability correlated with cell hydrophobicity (Zupancic et al., 2019).

Polyvinyl alcohol can also promote the viability of other probiotic bacteria, such as *Bifidobacterium lactis* Bb12 and *Streptococcus thermophilus* (TH-4). Together with *Lb. paracasei* 431, these bacteria remained viable after 1 week at room temperature (Akbar et al., 2018). *Bifidobacterium animalis* Bb12 also showed higher viability when incorporated into PVA nanofibers; this bacterium remained viable for 40 days at room temperature, and for 130 days at refrigeration temperatures (Lopez-Rubio et al., 2009).

Apart from therapeutic applications, nanofibers with incorporated lactobacilli can also be used for food preservation in food technology. Lactic acid bacteria convert sugar into lactic acid, which results in an acidic environment that can eliminate harmful bacteria. Incorporation of *Lb. rhamnosus* GG into PVA/alginate nanofibers enhanced the viability of this bacterium and has been used for the preservation of fish filets. The incorporated bacteria delayed the growth of total mesophilic aerobic bacteria and psychrophilic bacteria in fish filets by 38%, but did not reduce the growth of fungus and mold (Ceylan et al., 2018). Applications in food technology have also been suggested for *Lb. gasseri*, whereby its incorporation into PVA nanofibers promoted the viability of *Lb. gasseri* over 1 month at 4°C (Amna et al., 2013).

The stability of probiotics and other microorganisms can be enhanced by the production of biofilms. Biofilms represent an alternative lifestyle for microorganisms, which allow them to adopt a multicellular behavior and to prolong their survival under adverse conditions. In biofilms, the microorganisms are protected by the secretion of extracellular matrix composed polysaccharides and proteins. Electrospun cellulose acetate nanofibers have been used as scaffolds for single-species *Lb. plantarum* biofilms. The probiotic biofilms on these nanofibers were resistant to the gastrointestinal environment, and the *Lb. plantarum* survived longer than planktonic *Lb. plantarum*. After 3 weeks of storage, 27.40% of biofilm cells survived, in contrast to only 0.77% of planktonic cells (Hu et al., 2019).

Lactobacilli and bifidobacteria can also be found in the oral cavity; however, their role in oral health is questionable due to their ability to decrease the pH, which can lead to tooth decay. *Bacillus* spp. 25.2M is also present in the oral cavity, and can produce antibacterial substances. Furthermore, this bacterium can overgrow the periodontal pathogen *Aggregatibacter actinomycetemcomitans in vitro*, which makes it a promising probiotic candidate. *Bacillus* spp. 25.2M cells did not survive the electrospinning conditions and thus its spores were incorporated into chitosan and PEO nanofibers. The viability of spores decreased by a maximum of one log unit during electrospinning, but it remained almost unchanged during 1 year storage of nanofibers. This indicates that these nanofibers represent a promising delivery system in the treatment of periodontal disease and for restoration of the oral microbial balance (Zupancic et al., 2018).

In diabetic patients, alterations to the skin microbiota represent a promising strategy to prevent diabetic foot ulcers. The pathogen *Staphylococcus aureus* can colonize the skin, and can cause serious damage to the tissue. On the other hand, *S. epidermidis* is a representative of normal skin microbiota, and it can inhibit the growth of pathogens through production of antimicrobial substances, and induction of epithelia growth. Carboxymethyl cellulose together with PEO was used to produce a nanofiber delivery system for *S. epidermidis* that allowed its delivery to the skin microbiome in a viable form and that can serve as an alternative in the treatment of diabetic foot ulcers (Kurecic et al., 2018).

Biosensors can detect and identify a component within a cell or a tissue. The Gram-negative aerobe *Gluconobacter oxydans* has been used as a biosensor for glucose (i.e. gluconosensor). The oxidative enzymes of *Gluconobacter oxydans* allow for the oxidation of glucose to gluconic acid, and the consequent reduction of hexacyanoferrate (III). Formed hexacyanoferrate (II) is re-oxidized at an electrode and this reaction is monitored amperometrically (Aslan and Anik, 2016). Immobilization of *Gluconobacter oxydans* into PCL/PEI nanofibers facilitated oxygen and glucose transfer to the cells, which resulted of shorter sensor response time, and also decreased the limit of detection for glucose (Gordegir et al., 2019).

Electrospun nanofiber delivery systems can also be of great use in agricultural engineering for seed inoculation. Rhizobacteria are a group of microorganisms that colonize many plant species. They can protect the plants from environmental stress and increase the nutrient uptake. However, their viability during seed treatments and storage represents a major problem. The incorporation of two rhizobacteria (*Pantoea agglomerans* ISIB55 and *Burkholderia caribensis* ISIB40) into PVA nanofibers increased their survival on soybean seeds, compared to the un-incorporated bacterial cultures, over the course of 30 days. The decreases in viability were ∼2 log units and ∼4 log units for incorporated and un-incorporated *Pantoea agglomerans* ISIB55, respectively; and ∼1.5 log units and ∼4 log units for incorporated and un-incorporated *Burkholderia caribensis* ISIB40. Additionally, *P. agglomerans* ISIB55 increased germination rates and length and dry weight of the roots, and *B. caribensis* ISIB40 increased the leaf numbers and dry weight of the shoots (De Gregorio et al., 2017).

Cyanobacteria are photosynthetic organisms that are sometimes also classified as algae. They are used as food supplements, as they contain vitamins, minerals, and fatty acids. *Spirulina* is a genus of cyanobacteria that is most commonly used in food supplements. Its incorporation into nanofibers has been suggested for tissue engineering, treatment of spinal cord injuries, and as an antibacterial therapy. In contrast to other bacteria where live cells are incorporated into nanofibers, *Spirulina* was initially processed (i.e. dried, milled) and its biomass was used in the electrospinning. The application of PHB–hydroxyvalerate and PCL nanofibers that contained *Spirulina* sp. LEB 18 biomass as the bioactive component was suggested for the field of tissue regeneration. *Spirulina* sp. LEB 18 improved the physical characteristics of the nanofibers, and provided the nanofiber scaffold with reduced healing times and stimulated cell growth (Schmatz et al., 2016). *Spirulina* biomass was also incorporated into PEO nanofibers, which were suggested as an extracellular matrix for stem-cell culture and future treatment of spinal cord injury (de Morais et al., 2010). One of the characteristics of *Spirulina* is that it can produce polymers that can themselves be made into nanofibers. Biopolymer PHB is produced by *Spirulina* sp. LEB 18, and this was used to make nanofibers that additionally contained cyanobacterial phenolic compounds that have antibacterial, antifungal, and antioxidant activities. These nanofibers inhibited the growth of *Staphylococcus aureus* ATCC 25923, and they were suggested for use in food packaging (Kuntzler et al., 2018).

### Fungi

The incorporation of fungi into nanofibers has focused on the exploitation of their biocatalytic properties, rather than their biotherapeutic properties, as shown in Table 2. *Candida tropicalis* is one of the most virulent of the *Candida* species that can cause several human diseases. However, their degradation of a wide range of environmental pollutants has increased their use for purification of waste waters. Water-soluble nanofibers cannot be used in aqueous environments, such as wastewaters, and therefore coaxial electrospinning has been used to form nanofibers with a water-soluble core and a hydrophobic shell. *C. tropicalis* has been successfully encapsulated into nanofibers composed of a PVP core and a polyvinylidene fluoride plus hexafluoropropylene shell using coaxial electrospinning. The incorporated fungi degraded phenols and fermented ethanol in olive-mill wastewater, and also showed toxicity against *E. coli* (Letnik et al., 2015). Other types of fungi that have been suggested for purification of waters include *Kluyveromyces lactis* and *Saccharomyces cerevisiae*. When these two heat-inactivated fungi were immobilized in PVA and cellulose acetate hybrid nanofibers, they provided enhanced removal of aflatoxin B2 from contaminated water by binding it to their surface. The resulting water was less cytotoxic for human fibroblasts (Moustafa et al., 2017). Incorporation of *S. cerevisiae* EBY100 into PVA nanofibers preserved its viability after electrospinning, which suggested potential biocatalytic applicability (Canbolat et al., 2013). *Trichoderma viride* spores were incorporated into electrospun nanofibers composed of PEO, polyacrylamide and chitosan, and its viability was preserved. The incorporated fungus spores inhibited the growth of phytopathogenic strains (i.e. *Fusarium*, *Alternaria*) (Spasova et al., 2011).

**TABLE 2:** Systems for incorporation of fungi into electrospun nanofibers, and their designed uses.

**Fungus**	**Nanofiber polymer**	**Purpose**	**References**
*C. tropicalis*	Polyvinyl pyrrolidone/polyvinylidene fluoride + hexafluoropropylene	Wastewater treatment (biocatalysis)	Letnik et al., 2015
*K. lactis*	Polyvinyl alcohol/cellulose acetate	Wastewater treatment (aflatoxin binding)	Moustafa et al., 2017
*S. cerevisiae*			
*S. cerevisiae* EBY100	Polyvinyl alcohol	Biocatalysis	Canbolat et al., 2013
*T. viride*	Chitosan/polyethylene oxide/polyacrylamide	Agriculture (plant protection)	Spasova et al., 2011

### Stem Cells

Stem cells can differentiate into other cell types and thus build any tissue in the body. These characteristics mean that they can have a variety of therapeutic benefits, and can be used for different purposes. However, stem cells have limited viability and are difficult to proliferate, which impedes their wider use for a variety of potential therapeutic benefits. Combinations of stem cells and electrospun nanofibers have two main advantages. First, the nanofibers can serve as a favorable scaffold for maintenance of the stem cells through alteration of the chemical properties of the nanofibers in such a way as to improve their interactions with the stem cells. Secondly, nanofibers can serve as a system for delivery of stem cells into specific tissues or organs for tissue engineering and wound healing. PLGA is the most common polymer that has been used for the production of nanofibers for stem cells. PLGA is a biocompatible polymer that supports the growth of stem cells as well as their multi-lineage differentiation (Rowland et al., 2016). Stem-cell types that have been seeded onto such nanofiber scaffolds have included MSCs (derived from bone marrow) and adipose-derived stem cells (Table 3). Of these, each type has various potential clinical applications (Strioga et al., 2012). For example, together with other cell lineages (e.g. fibroblasts, keratinocytes, endothelial cells), MSCs have very important roles in wound healing. MSCs can stimulate the formation of new blood vessels, modulate inflammatory responses, promote migration of keratinocytes, and improve extracellular matrix production (Tartarini and Mele, 2016).

**TABLE 3:** Systems for incorporation of stem cells into electrospun nanofibers, and their designed uses.

**Stem cell**	**Nanofiber polymer**	**Purpose**	**References**
Adipose-derived stem cells + platelet-derived growth factor BB	Poly (lactic-co-glycolic acid)	Tissue regeneration (tendon repair)	Manning et al., 2013
Mesenchymal stem cells	Poly (lactic-co-glycolic acid)/chitosan	Tissue regeneration (spinal cord injury)	Kim Y. C. et al., 2016
	Poly (lactic-co-glycolic acid)	Immuno-regulation (arthritis)	Zhang et al., 2014
	Collagen/poly (L-lactic acid)–co–polycaprolactone	Tissue regeneration (wound healing)	Jin et al., 2011
Adipose-derived stem cells	Poly L-lactic acid	Compatibility testing	Xavier et al., 2016

Growth of stem cells on nanofibers has positive impact on their viability. Poly L-lactic acid nanofibers have been shown to be compatible with MSCs, as these cells showed high cell viability and high rates of proliferation, while their normal morphological characteristics were maintained (Xavier et al., 2016). Adipose-derived stem cells were combined with platelet-derived growth factor BB (PDGF-BB) and incorporated into a heparin/fibrin-based delivery system hydrogel, which was then layered with PLGA nanofibers. The seeded cells remained viable and PDGF-BB showed sustained release. The nanofiber scaffold was thus suggested as a potential delivery system for tendon repair (Manning et al., 2013). MSCs seeded on PLGA and chitosan nanofibers have shown better cell engraftment and neuroprotective effects in rats with spinal cord injury compared to intra-lesional injection of MSCs (Kim Y. C. et al., 2016). MSCs have also been used for treatment of autoimmune diseases, due to their immunoregulatory actions. MSCs seeded on PLGA nanofibers suppressed T-cell proliferation and inhibited systemic inflammatory reactions, which led to suppression of arthritis and bone destruction (Zhang et al., 2014). MSCs have also been successfully grown on a collagen/poly (L-lactic acid)–co–polycaprolactone nanofiber scaffold, where they differentiated into keratinocytes and expressed keratin 10, filaggrin, and involucrin (Jin et al., 2011).

### Viruses

Viruses cannot undergo independent life; however, they show four fundamental characteristics of living organisms: multiplication, genetic information, mutation, and evolution (Pennazio, 2011). Although viruses are generally regarded as pathogenic organisms, they can have therapeutic benefits when administered in the correct tissue or organ at the correct dose. Incorporation of viruses into nanofibers can maintain their viability over long periods. Nanofibers with incorporated viruses can serve as an alternative delivery vehicle for viruses, and can be used as a therapy against bacteria or cancer, as well as for gene delivery (Table 4).

**TABLE 4:** Systems for incorporation of viruses into electrospun nanofibers, and their designed uses.

**Virus**	**Nanofiber polymer**	**Purpose**	**References**
Bacteriophage vB_Pae_Kakheti25	Polycaprolactone	Antibacterial (textile protection)	Nogueira et al., 2017
Bacteriophages T7, T4, λ	Polyvinyl alcohol	Antibacterial (phage therapy)	Salalha et al., 2006
Adeno-associated virus	Elastin-like polypeptides/polycaprolactone	Gene delivery	Lee et al., 2011
Adenovirus	Polycaprolactone	Gene delivery	Liao et al., 2009
Vaccinia virus	Poly (lactic-co-glycolic acid)	Anticancer (colon cancer therapy)	Badrinath et al., 2018

Viruses that have antibacterial effects are called bacteriophages. The vB_Pae_Kakheti25 bacteriophage capsid immobilized into PCL electrospun nanofibers showed antibacterial effects against *Pseudomonas aeruginosa*, a pathogen that causes acute and chronic skin infections (Nogueira et al., 2017). The bacteriophages T7, T4, and λ maintained their viability when they were encapsulated in PVA nanofibers over 3 months at −4°C, −20°C, and −55°C (Salalha et al., 2006).

Vaccinia virus has oncolytic activity against colorectal cancer; however, its administration to humans is still challenging due to its immunogenicity and its spread to other organs, thus causing health problems. Vaccinia virus incorporated in PLGA electrospun nanofibers was shown to be a promising alternative for local delivery of the virus, and has demonstrated enhanced apoptosis of colon cancer cells (Badrinath et al., 2018).

Viruses allow transduction of certain cells with nucleic acids, which can result in gene delivery or therapy; the latter can be improved by the use of an effective virus delivery system. Adeno-associated virus is a safe non-pathogenic virus, and it represents a highly efficient vehicle for the delivery of specific genes. Adeno-associated virus encapsulated in nanofibers composed of a mixture of elastin-like polypeptides and PCL was shown to promote effective transduction of fibroblast cells (Lee et al., 2011). Efficient transduction of the green fluorescent protein (GFP) gene was also achieved in HEK 293 cells when they were cultured on PCL nanofibers containing engineered adenovirus (Liao et al., 2009).

## Proteins and Nucleic Acids in Nanofibers

The majority of the proteins and nucleic acids that have been incorporated into nanofibers were intended for therapies, as biopharmaceuticals. Protein biopharmaceuticals include hormones, growth factors, enzymes, and antibodies, while nucleic-acid biopharmaceuticals include plasmid DNA, DNA fragments, and RNA. Due to their sensitive molecular structures, the design of efficient delivery systems for protein and nucleic-acid biopharmaceuticals is necessary to protect the drug, as well as to obtain the desired drug concentration at the specific tissue or organ. Other applications of nanofibers with proteins and nucleic acids include biocatalysis and biosensing.

### Proteins

Proteins have wide-ranging applications in medicine as therapeutics for several chronic conditions, such as anemia, infections, rheumatoid arthritis, cancers, diabetes, nerve injuries, chronic wounds, and others (Reynolds et al., 2013). Their most common route of administration is parenteral, which has several disadvantages, such as the short half-life *in vivo*, frequent injections, and poor patient compliance (Patel et al., 2014). Other routes of administration are challenging, because protein molecules are large in size and are susceptible to proteolysis and low pH, which results in poor absorption and poor therapeutic efficacy. The development of appropriate carrier systems for efficient protein delivery is therefore important, and electrospinning of proteins into different natural and synthetic polymers can offer attractive alternatives to current methods (Table 5).

**TABLE 5:** Systems for incorporation of proteins into electrospun nanofibers, and their designed uses.

**Protein**	**Nanofiber material**	**Purpose**	**References**
Insulin	Polyvinyl alcohol/sodium alginate	Diabetes treatment (transmucosal delivery)	Sharma et al., 2013
	Chitosan/polyethylene oxide	Diabetes treatment (transbuccal delivery)	Lancina et al., 2017
	Fish sarcoplasmic proteins	Diabetes treatment (oral delivery)	Stephansen et al., 2015b
Peroxidase and alkaline phosphatase	Eudragit L100	Simulating oral enzyme delivery	Frizzell et al., 2017
PDGF−BB	Polyethylene oxide/polycaprolactone	Bone tissue regeneration	Briggs and Arinzeh, 2014
Growth hormone	Eudragit L100/chitosan	Oral mucositis treatment	Choi et al., 2016
EGF	Silk/polyethylene oxide	Chronic non-healing wounds treatment	Schneider et al., 2009
Glial cell-derived neurotrophic factor	Polycaprolactone-co-ethyl ethylene phosphate	Nerve regeneration	Chew et al., 2007
Nerve growth factor	Polycaprolactone-co-ethyl ethylene phosphate	Nerve regeneration	Chew et al., 2005
Nerve growth factor + monosialoganglioside	Poly (L-lactic acid-co-caprolactone)/silk fibroin	Simulating cell proliferation and differentiation	Sun et al., 2016
Vascular endothelial growth factor	Polyethylene carbonate-ε-caprolactone	Simulating cell proliferation and adherence	Zhang et al., 2012
Lysozyme	Poly (DL-lactide)/methyl cellulose	Simulating enzyme release	Yang et al., 2008
Lipase from *Candida rugosa*	Polyvinyl alcohol	Biocatalysis	Wang and Hsieh, 2008
Bovine serum albumin	Polyethylene oxide	Biosensing (pH)	Kowalczyk et al., 2008

Diabetes is a metabolic disease with increasing incidence, especially in developed countries, due to the trends of urbanization, and changes in lifestyle and environment. Insulin is central in diabetic treatment, and its safe and comfortable delivery is favored, where the oral route, if possible, would be the most preferred. Biodegradable PVA and sodium alginate electrospun nanofibers can provide sustained and controlled release of insulin with first-order kinetics that follows an initial burst release. Antidiabetic properties have been reported for male rats when insulin nanofibers were administered sublingually (Sharma et al., 2013). The buccal mucosa is an attractive drug-delivery route due to the high permeability and vascularization; however, the permeability is still limited by tight junctions between the epithelial cells. Chitosan can be used to loosen these junctions, thus allow diffusion to take place. Chitosan nanofibers blended with PEO have been tested for transbuccal insulin delivery, with 16-fold greater buccal permeability seen compared to free insulin (Lancina et al., 2017). To enable successful oral insulin delivery, the acidic pH in the stomach and the presence of proteolytic enzymes in the small intestine would also have to be overcome. Nanofibers made from fish sarcoplasmic proteins have been shown to protect insulin against chymotrypsin degradation, and to facilitate the opening of tight junctions of Caco-2 cell monolayers, thus providing increased transportation of around 12% of insulin without compromising cell viability (Stephansen et al., 2015a).

Growth factors are biologically active proteins that are secreted, and they affect the growth of cells. Among other uses, they can be administered for nerve regeneration, wound healing, and cell proliferation. Encapsulation of neurotrophin-3 in nanofibers composed of PCLEEP and collagen prolonged neurotrophin-3 availability and demonstrated its controlled release over a period of 2 months. Furthermore, neurotrophin-3 incorporated into these nanofibers stimulated axon regeneration in female rats 1 week post implantation (Nguyen et al., 2017). Coaxial electrospinning of poly (L-lactic acid)–co–polycaprolactone as the shell, and bovine serum albumin together with nerve growth factor (NGF) as the core, effectively promoted nerve regeneration in rats (Liu et al., 2011). PCLEEP has also served for the incorporation of other neurotrophic factors as well as NGF, such as human glial cell-derived neurotrophic factor, which was incorporated into PCLEEP nanofibers and enabled 44% recovery of electrophysiological responses in rats (compared to the control empty nanofiber scaffold, with no recovery seen) (Chew et al., 2007). NGF has a short half-life *in vivo* (<5 h), while its encapsulation into PCLEEP nanofibers provided its sustained release over 3 months. This encapsulated NGF also retained its bioactivity, as seen by stimulation of PC12 cell differentiation into neurons (Chew et al., 2005). Furthermore, poly (L-lactic acid-co-caprolactone)/silk fibroin blend nanofibers that contained the combination of NGF and monosialoganglioside promoted increased cell differentiation and proliferation compared to nanofibers with only NGF incorporated (Sun et al., 2016).

Wounds can be very problematic for patients, and can have life-threatening complications. Many cancer patients suffer from oral mucositis, which is caused by chemotherapy and radiotherapy, and the lack of a functional delivery system in their treatment can make this situation worse. Human growth hormone incorporated into nanofibers composed of Eudragit L 100 (methacrylic acid, methyl methacrylate) and chitosan enabled full recovery and regeneration of the cellular epithelium after 7 days of treatment of dogs with oral mucositis (Choi et al., 2016). EGF is a protein that promotes wound healing by stimulation of cell proliferation and migration of keratinocytes. The application of EGF in wound healing is limited, however, due to its short half-life of about 1 h, and thus the need for frequent administration to the desired area. EGF incorporated into silk and PEO nanofibers showed slow release (25% in 170 h), which resulted in almost full wound closure after 48 h (Schneider et al., 2009). PEO with PCL have also been used to incorporate PDGF−BB using emulsion electrospinning, with the addition of the non-ionic surfactant Span80 and mild sonication. The nanofibers prepared in this way provided controlled release of PDGF-BB over the course of 96 h, and enhanced the osteogenic differentiation of human MSCs (Briggs and Arinzeh, 2014). Incorporation of angiogenesis-stimulating vascular endothelial growth factor into biodegradable polyethylene carbonate-ε-caprolactone electrospun nanofibers also promoted the growth and viability of human umbilical vein endothelial cells, and has thus been suggested as a promising delivery system to stimulate blood vessel formation (Zhang et al., 2012).

The stability and bioavailability of enzymes can also be improved by their incorporation into nanofibers. Encapsulation of lysozyme in poly (DL-lactide) and methyl cellulose nanofibers using emulsion electrospinning protected the structural integrity and bioactivity of lysozyme. Furthermore, its release was modified such that it showed sustained release over 2 weeks (Yang et al., 2008). Horseradish peroxidase and alkaline phosphatase have been incorporated into Eudragit L100 nanofibers using an emulsion electrospinning technique, which provided controlled release based on the pH of the environment. At acidic pH, Eudragit L100 was insoluble, and showed <5% enzyme release. Under neutral and basic conditions, Eudragit L100 was ionized and was dissolved, whereby at pH 6 it showed ∼100% enzyme release within 1 h. This system has been suggested to allow safe delivery of these enzymes to the gastrointestinal track by avoiding the acidic conditions in the stomach (Frizzell et al., 2017). PEO is another nanofiber material that is sensitive to the pH of the environment, as it releases its ‘payload’ at alkaline pH. PEO nanofibers loaded with bovine serum albumin and labeled with a fluorescein isothiocyanate have been used as pH biosensors, and also for visualization of nanofibers and their interactions with cells (Kowalczyk et al., 2008). Finally, a lipase from *Candida rugosa* that is frequently used in biocatalytic transformations has been successfully immobilized in PVA nanofibers, wherein it showed greater stability under different environment conditions (85% activity after 4 h incubation at 40°C) compared to the soluble enzyme (30% activity after 1 h incubation at 40°C; Wang and Hsieh, 2008).

### Nucleic Acids

Nucleic acids (i.e. DNA, RNA) are charged compounds with large molecular weights that have been recognized as therapeutic agents for different diseases, such as neurodegenerative diseases, cancers, and immunological disorders, among others. Although they have great therapeutic potential, their delivery still represents a challenge due to their instability in biological environments, and their immunogenicity and toxicity (Sridharan and Gogtay, 2016).

Nanofibers can be used for delivery of plasmid DNA, miRNAs, and small-interfering (si)RNAs (Table 6). Plasmid DNA can be incorporated in nanofibers for non-viral gene delivery. Plasmid pCMVβ, which encodes β-galactosidase, was successfully incorporated into PLGA and PLA–PEG electrospun nanofibers. The incorporated plasmid was taken up by MC3T3 cells, and promoted their expression of the β-galactosidase gene. Adding equivalent amounts of the non-incorporated plasmid to the cells resulted in no cellular transfection (Luu et al., 2003). The same plasmid containing the GFP gene and incorporated into a PLA–PEG–PLA triblock co-polymer also transfected MC3T3 cells (Liang et al., 2005). Coaxial nanofibers of PCL and PEG as the sheath and core, respectively, were used to deliver the plasmid pCMV-eGFP that encodes enhanced (e)GFP, over 60 days. The transfection of CRL 1764 fibroblasts was improved by adding PEI–hyaluronic acid to the sheath (Saraf et al., 2010).

**TABLE 6:** Systems for incorporation of nucleic acids into electrospun nanofibers, and their designed use.

**Nucleic acid**	**Nanofiber material**	**Purpose**	**References**
Plasmid (β-galactosidase gene)	Poly (lactic-co-glycolic acid) + poly (lactic-co-glycolic acid)/polyethylene glycol	Simulating gene delivery	Luu et al., 2003
Plasmid (eGFP gene)	Polycaprolactone/polyethylene glycol	Simulating gene delivery	Saraf et al., 2010
	Poly (DL-lactide)-poly (ethylene glycol)	Simulating gene delivery	Yang et al., 2011
	Polycaprolactone	Simulating gene delivery	Ceylan et al., 2013
	Poly(1,4-butanediol diacrylate-co-4-amino-1-butanol) end-capped with 1-(3-aminopropyl)-4-methylpiperazine	Simulating gene delivery	Li et al., 2013
Plasmid (β-galactosidase/GFP genes)	Polylactic acid/polyethylene glycol/poly (lactic-co-glycolic acid)	Simulating gene delivery	Liang et al., 2005
Plasmids (vascular endothelial growth factor/fibroblast growth factor genes)	Poly (DL-lactide)-poly (ethylene glycol)	Gene delivery (regeneration of blood vessels)	Chen et al., 2013
MicroRNA (miRNA-222)	Polycaprolactone-co-ethyl ethylene phosphate	Gene silencing of *NTF3*	Nguyen et al., 2017
MicroRNAs (miRNA-219, miRNA-338)	Polycaprolactone	Gene silencing of *PDGFR*-α, *Sox6*, *Hes5*, *FoxJ3*, *ZFP238*	Diao et al., 2015
siRNA	Polycaprolactone or polycaprolactone/polyethylene glycol	Gene silencing of *GAPDH*	Cao et al., 2010
	Polycaprolactone-co-ethyl ethylene phosphate	Gene silencing of *GAPDH*	Rujitanaroj et al., 2011

As for coaxial electrospinning, emulsion electrospinning has also been used to make core–sheath nanofibers for delivery of plasmid DNA. Here, the core contained plasmid DNA (eGFP-N2, encoding GFP), without and with PEI, while the sheath was made of poly (DL-lactide)-poly (ethylene glycol) without and with PEG or PEI. Sustained release of DNA was obtained between 6 days and 25 days, and efficient transfection of NIH3T3 cells was achieved (Yang et al., 2011).

Polycaprolactone nanofibers containing the GFP-encoding plasmid pCMVβ were used for transfecting fibroblasts. The greatest release of the plasmid was seen in the first 15 min, which was followed by sustained release for more than 1 week (Ceylan et al., 2013). Another DNA release system was based on nanofibers composed of poly (β-amino ester) poly (1,4-butanedioldiacrylate-co-4-amino-1-butanol) that was end-capped with 1-(3-aminopropyl)-4-methylpiperazine; this system provided sustained release of DNA for more than 24 h, and the nanofibers enhanced transfection of the GB 319 human glioblastoma cell line (Li et al., 2013).

These nanofibers that contained GFP-encoding plasmids have been used as proof-of-principle to demonstrate effective gene delivery. However, nanofibers have also been used to deliver plasmids for specific therapeutic purposes. For example, plasmids that encoded vascular endothelial growth factor and basic fibroblast growth factor have been loaded into calcium phosphate nanoparticles, and electrospun into poly (DL-lactide)-poly (ethylene glycol) nanofibers. The plasmids were released over 4 weeks, and successfully transfected into endothelial and smooth muscle cells. This resulted in the formation of a vascular network and rapid generation of mature blood vessels (Chen et al., 2013).

DNA is not the only nucleic acid that can be used for therapeutic purposes. RNA molecules (i.e. miRNAs, siRNAs) can regulate expression of certain genes, such as those encoding NTF3, GAPDH, PDGFR-α, Sox6, Hes5, FoxJ3, and ZFP238, thus improving the health of the patients. miRNA-222 is a non-coding RNA that is involved in local protein synthesis and enhances axonal re-growth. The previously mentioned nanofibers that were composed of PCLEEP and collagen were used to incorporate micellar nanoparticles that contained miRNA-222. A total of 27.1% miRNA was released within the first month, followed by 2 months of steady release. The delivery of the miRNA supported axon regeneration in mice for 10 days post-implantation (Nguyen et al., 2017). miRNA-219 and miRNA-338 inhibit the expression of negative regulators of differentiation of oligodendroglial precursor cells, such as PDGFR-α, Sox6, Hes5, FoxJ3, and ZFP238. This might have positive effects against central-nervous-system–related diseases, and more precisely in oligodendrocyte differentiation and maturation. Incorporation of these miRNAs into PCL nanofibers efficiently knocked-down these inhibitory regulators, and thus enhanced differentiation of oligodendroglial precursor cells to oligodendrocytes, in 4–7 days (Diao et al., 2015).

Small-interfering RNAs are double-stranded RNA molecules, and like miRNAs, they can regulate gene expression. Glyceraldehyde 3-phosphate dehydrogenase (GAPDH) is involved in glycolysis, and is often used as a model (‘housekeeping’) gene. GAPDH siRNA was encapsulated in PCL and PCL/PEG nanofibers and successfully transfected into HEK 293 cells. This siRNA was released from the nanofibers over at least 28 days, with high GAPDH silencing efficiency seen (61–81%) (Cao et al., 2010). Similar data were obtained by Rujitanaroj et al. (2011), who encapsulated GAPDH siRNA together with transfection reagent complexes in PCLEEP electrospun nanofibers. The GAPDH siRNA both without and with the transfection reagent complexes was detected for up to 28 days. The silencing efficiency of this GAPDH siRNA system with transfection reagent complexes was 58%, in comparison to 40% without transfection reagent complexes (Rujitanaroj et al., 2011).

## Conclusion

Electrospinning is an established method for the formation of nanofibers; i.e. nanomaterials that appear under the microscope as a polymeric nanofiber mesh, and that macroscopically appear as a thin plastic film. Nanofibers are formed from a polymer solution, which allows straightforward incorporation of chemicals by simply mixing them into the solution. Biological products, such as live cells, proteins, and nucleic acid, are sensitive to pH, temperature, and enzymatic degradation. Therefore, their incorporation into nanofibers using electrospinning might appear counterintuitive, particularly due to the high voltage that is used during the electrospinning process. However, although still relatively rare, a considerable number of studies from the literature are reviewed here, and demonstrate that incorporation of live cells, viruses, functional proteins, and nucleic acids into nanofibers is feasible and advantageous. The rationale behind the incorporation of biological products is their protection from environmental influences (i.e. to improve their viability or stability), and their immobilization, drying and subsequent solidification for the formation of a flexible and effective form of delivery. The majority of nanofibers that contain biological products are intended as an alternative delivery vehicle for use in medical therapies, whereby they provide protection and a growth scaffold, and actively improve delivery (e.g. transfection of nucleic acids or growth of stem cells), with the added advantage of a large surface-to-volume ratio.

Both natural and synthetic polymers have been used to make nanofibers. PVA, PEO, cellulose and chitosan polymers are most frequently used for incorporation of bacteria and fungi. Viruses have usually been incorporated into PCL nanofibers, and stem cells grown on PLGA nanofibers. These materials differ in their hydrophobicity and hydrophilicity, and under physiological conditions this can allow sustained or rapid release, respectively. Alternatively, pH responsive polymers (e.g. Eudragit) have been suggested for controlled release under the conditions of the gastrointestinal tract.

To summarize, the literature reviewed here clearly demonstrates that incorporation of biological products into electrospun nanofibers is feasible and functional. However, most studies have focused on proof-of-principle experiments and the incorporation of model biological products. More studies are needed to consolidate the industrial applicability of electrospinning of biological products, particularly in the field of medical therapies.

## Author Contributions

SS wrote the draft of the manuscript. AB wrote and revised the manuscript. SS and AB approved the submitted version.

## Conflict of Interest

The authors declare that the research was conducted in the absence of any commercial or financial relationships that could be construed as a potential conflict of interest.

## Footnotes

1 https://www.genengnews.com/a-lists/top-15-best-selling-drugs-of-2018/

## Abbreviations

EGF: epidermal growth factor
eGFP: enhanced green fluorescent protein
GAPDH: glyceraldehyde 3-phosphate dehydrogenase
miRNA/: micro-RNA
MSCs: mesenchymal stem cells
NGF: nerve growth factor
PCL: poly (ε-caprolactone)
PCLEEP: poly (ε-caprolactone -co-ethyl ethylene phosphate)
PDGF: platelet-derived growth factor
PEG: poly (ethylene glycol)
PEI: polyethylenimine
PEO: polyethylene oxide
PHB: poly (hydroxybutyrate)
PLA: poly lactic acid
PLGA: poly (lactic-co-glycolic acid)
PVA: polyvinyl alcohol
PVP: polyvinylpyrrolidone
siRNA: small-interfering RNA.
